# In Situ Optical Spectroscopy Demonstrates the Effect of Solvent Additive in the Formation of All-Polymer Solar Cells

**DOI:** 10.1021/acs.jpclett.2c03397

**Published:** 2022-12-13

**Authors:** Yanfeng Liu, Qunping Fan, Heng Liu, Ishita Jalan, Yingzhi Jin, Jan van Stam, Ellen Moons, Ergang Wang, Xinhui Lu, Olle Inganäs, Fengling Zhang

**Affiliations:** † Biomolecular and Organic Electronics, Department of Physics, Chemistry and Biology, Linköping University, Linköping SE-581 83, Sweden; ‡ College of Materials and Textile Engineering, Nanotechnology Research Institute, Jiaxing University, Jiaxing 314001, China; § Department of Chemistry and Chemical Engineering, Chalmers University of Technology, Göteborg SE-412 96, Sweden; ∥ State Key Laboratory for Mechanical Behavior of Materials, Xi’an Jiaotong University, Xi’an 710049, China; ⊥ Department of Physics, The Chinese University of Hong Kong, Shatin 999077, Hong Kong, China; # Department of Engineering and Chemical Sciences, Karlstad University, Karlstad SE-651 88, Sweden; ∇ China-Australia Institute for Advanced Materials and Manufacturing, Jiaxing University, Jiaxing 314001, China; ○ Department of Engineering and Physics, Karlstad University, Karlstad SE-651 88, Sweden

## Abstract

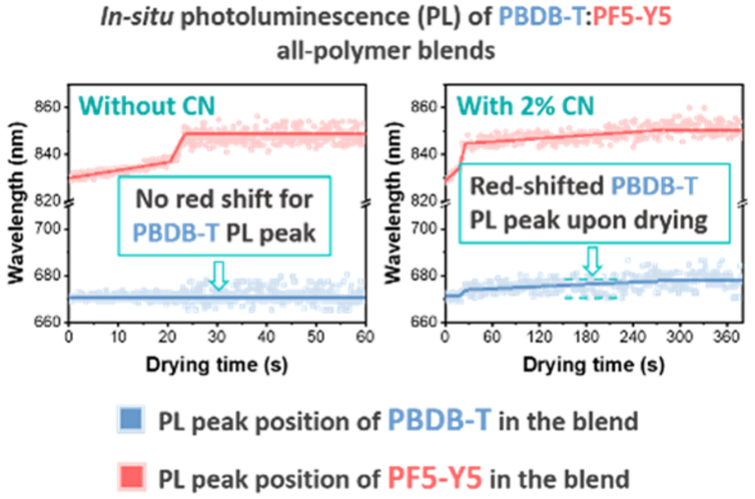

1-Chloronaphthalene (CN) has been a common solvent additive in both fullerene- and nonfullerene-based organic solar cells. In spite of this, its working mechanism is seldom investigated, in particular, during the drying process of bulk heterojunctions composed of a donor:acceptor mixture. In this work, the role of CN in all-polymer solar cells is investigated by in situ spectroscopies and ex situ characterization of blade-coated PBDB-T:PF5-Y5 blends. Our results suggest that the added CN promotes self-aggregation of polymer donor PBDB-T during the drying process of the blend film, resulting in enhanced crystallinity and hole mobility, which contribute to the increased fill factor and improved performance of PBDB-T:PF5-Y5 solar cells. Besides, the nonradiative energy loss of the corresponding device is also reduced by the addition of CN, corresponding to a slightly increased open-circuit voltage. Overall, our observations deepen our understanding of the drying dynamics, which may guide further development of all-polymer solar cells.

Within the rapid-growing field of photovoltaics, solution-processed organic solar cells (OSCs) have huge potential to bring solar energy into use at different scales, thanks to their superior compatibility with flexible substrates and roll-to-roll production.^1,2^ The typical photoactive layer of binary OSCs has a bulk-heterojunction (BHJ) structure consisting of a polymeric electron donor and a fullerene- or nonfullerene-based acceptor. OSCs based on nonfullerene acceptors (NFAs) these days outperform their fullerene-based counterparts, due to multiple advantages of NFAs including their more efficient light absorption in the visible and the near-infrared range, contributing to higher photocurrent, and their reduced nonradiative recombination losses, contributing to higher photovoltage.^3,4^ All-polymer solar cells (all-PSCs), consisting of both polymeric donor and acceptor materials, provide extra benefits compared to OSCs based on small molecular NFAs, including improved long-term operational stability and superior mechanical properties.^5,6^ Thus, all-PSCs may find promising applications in flexible and portable electronic devices.

Despite the impressive progress in power conversion efficiency (PCE) of state-of-the-art all-PSCs exceeding 18%,^7−10^ they still lag behind those of small-molecule-based NFA solar cells.^11,12^ The relatively lower PCEs of all-PSCs can be partly attributed to the limited availability of high-performance n-type polymers and the difficulty in controlling the morphology of the all-polymer blend films. The energetically favored polymer–polymer demixing can easily result in excessive phase separation,^13^ and the intricate chain entanglements make the proper BHJ morphology more difficult to acquire.^5^ Moreover, several donor polymers, including PBDB-T, have shown a strong tendency to aggregate at room temperature and disaggregate at higher temperatures in solution, as shown in temperature-dependent absorption spectroscopy experiments.^14,15^ Careful temperature control and processing conditions are therefore keys to controlling the competition between crystallization and phase separation and to realizing the desired photovoltaic performance of all-PSCs.

The use of solvent additives during solar cell fabrication has been proven as a convenient and efficient strategy to obtain improved BHJ morphology in OSCs.^16^ Two criteria for processing additives have been formulated, which served well in most fullerene-based OSCs: (a) selectively dissolve fullerene component and (b) higher boiling point than the main solvent.^17^ However, research showed that the experience of additive usage in fullerene-based systems sometimes cannot be simply copy-pasted into NFA-based systems.^18^ Therefore, the working mechanism of an additive has to be analyzed for each specific donor:NFA system. 1-Chloronaphthalene (CN) is a solvent with high boiling point (263 °C) that was introduced as a processing additive in many fullerene-based OSCs.^19−21^ It can also effectively enhance the performance of the device based on new Y-series small molecular NFA systems^22−24^ as well as the all-PSCs that are based on polymerized derivatives of the Y-series NFAs.^7,25,26^ However, the operating mechanism of CN during the drying process in these highly efficient all-polymer systems has not yet been thoroughly investigated. Thus, in this study in situ spectroscopy is employed to deepen the understanding of the effect of this additive during BHJ formation.

The donor–acceptor system used in this study is the polymer donor PBDB-T blended with the polymer acceptor PF5-Y5, which is a copolymer acceptor, originating from the Y-series acceptor Y5 and the thienyl-benzodithiophene unit.^7^ After proper optimization, this all-polymer blend showed more than 14% PCE when embedded into a conventional solar cell with the structure of ITO/PEDOT:PSS/PBDB-T:PF5-Y5/PDINO/Al. Specifically, the use of 2 vol/vol % CN (volume percent in the solution, denoted as 2% in the following discussion) as the solvent additive increased the device’s fill factor (FF) from 67.9% to 70.5%, also with an improved open-circuit voltage (*V*_OC_) from 0.922 to 0.948 V.^7^ Besides, a recent report shows how PF5-Y5 blend can be blade-coated from a number of different solvents and cosolvents and the correlation between these solvents/cosolvents and device performance.^27^ In this work, we focus on probing the mechanism of the solvent additive CN in forming this high-efficiency all-polymer BHJ. First, in situ photoluminescence (PL) and absorption spectroscopy are used to study the microstructure evolution during the drying of the active layer with or without CN. Detailed morphological studies of the resulting blend films with atomic force microscopy (AFM) and X-ray scattering-based techniques are carried out to gain a comprehensive view of the effect of CN from both in situ and ex situ perspectives. Based on our results, we find that the additive CN in the PBDB-T:PF5-Y5 system selectively promotes the aggregation of PBDB-T in the blends. This observation is different with some other all-polymer studies, where CN was found to act as a compatibilizer that prevents oversized aggregation of polymer with high crystallinity, like N2200.^28,29^ The distinct working mechanism of CN in different all-polymer systems highlights the necessity of updating our understanding regarding the role of additives in the newly developed materials for organic photovoltaics. Furthermore, the correlations between drying kinetics, film morphology, and charge transport properties as well as the device performance of the all-PSCs are elucidated.

The real-time evolution of the all-polymer BHJ films is recorded and investigated during blade coating with a combination of in situ PL spectroscopy, the laser scattering (LS) from the films, and in situ absorbance spectroscopy. Figure S1 shows the schematic diagrams of the setup used in this study. Figure 1a–d shows the evolution of the PL results of the polymer blends blade-coated in chlorobenzene (CB) solution and in CB with 2% of CN as a function of the drying time. The complete PL spectra can be found in Figure S2. The PL evolution of a pure component film of PBDB-T is described in our previous work,^30^ and the emission spectrum of PF5-Y5 film is presented in Figure S3a. The emission maximum of PBDB-T and PF5-Y5 is located around 670 and 840 nm, respectively. These well-separated emission peaks allow us to assign the peak at the shorter wavelength to PBDB-T and at the longer wavelength to PF5-Y5 in the PL spectra of blend films. As seen in Figure 1a,b, the emission peaks around 840 nm that correspond to PF5-Y5 in the blend film undergo spectral shifts during the film drying (red line). By defining the starting point of drying as **t0** and the time of spectral shift as **t1**, **t2**, ..., the complete blend drying can be divided into multiple stages.

**Figure 1:**
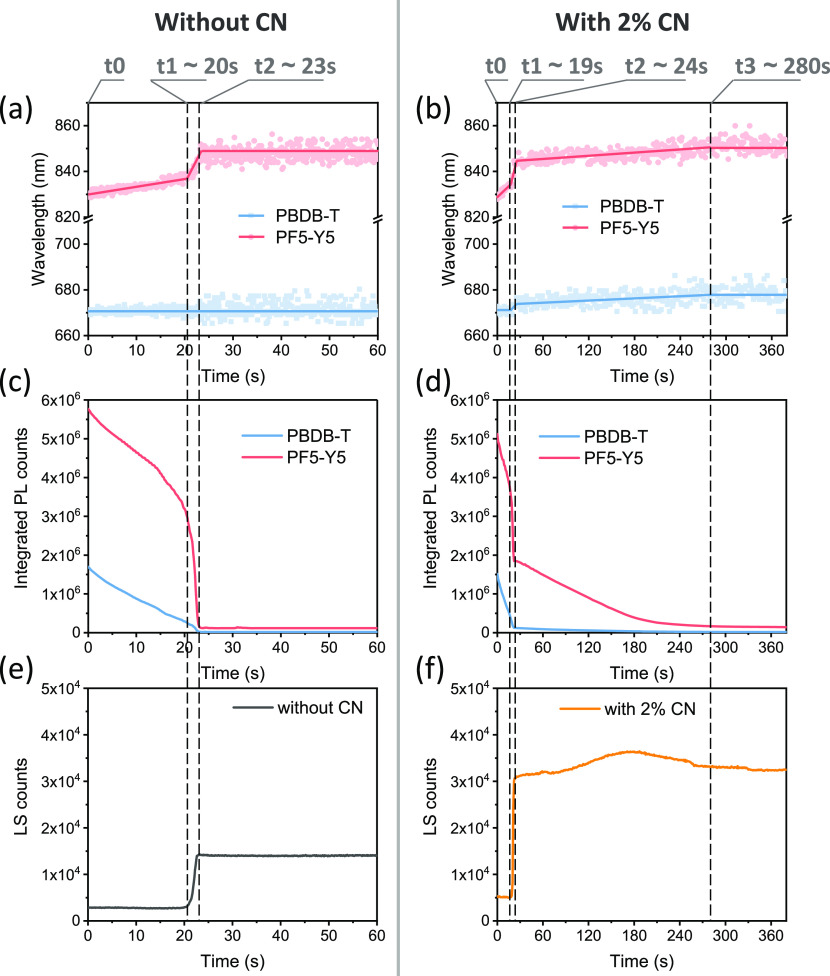
In situ PL and LS results of the PBDB-T:PF5-Y5 blend film blade-coated from CB without and with CN, as a function of drying time. (a, b) Locations of PBDB-T and PF5-Y5 emission peaks. (c, d) Peak intensity for PBDB-T and PF5-Y5 emission peaks (at 670 and 830 nm, respectively). (e, f) LS intensity of the blend with or without CN. The excitation wavelength is 532 nm.

For the blend without CN (Figure 1a,c,e), three drying stages can be identified: (1) the liquid film stage (**t0** < **t** < **t1**), where the emission from both PBDB-T and PF5-Y5 undergo a gradual quenching with a weak red-shift of the PF5-Y5 emission around 830 nm; (2) the liquid–solid transition stage (**t1** < **t** < **t2**), where the PBDB-T and PF5-Y5 show a more rapid quenching, accompanied by a rapid red shift of PF5-Y5 emission from 835 to 850 nm, and an increased LS signal; (3) the solid film stage (**t2** < **t**), where all the parameters including PL intensities, peak positions, and LS signal are constant. It is worth noting in Figure 1a that no spectral shift is observed for the PL peak at 670 nm, corresponding to PBDB-T, during the entire drying process. This is unexpected since our previous work clearly demonstrated that the emission peak maximum of pristine PBDB-T exhibited ca. 14 nm red shift from 672 to 686 nm between **t1** and **t2** in its drying process, and a red-shifted emission was also be observed in the blends where PBDB-T was mixed with acceptor polymer N2200 or small molecule acceptors.^30^ This indicates that the presence of PF5-Y5 in the blend hinders this red shift of the PBDB-T emission maximum. Similar to the PL results, the in situ absorbance measurement of the as-casted PBDB-T:PF5-Y5 blend (Figure S4a) does not show any red-shift of the PBDB-T absorption maximum either, which can be observed, however, during drying of pure PBDB-T films in our previous work^30^ and in Figure S3b (from 619 to 628 nm). Since such a spectral red-shift can be interpreted as a sign of polymer aggregation, we suggest that the absence of the red-shift for the PBDB-T emission and absorption peak indicates that the self-aggregation of PBDB-T is hindered during the liquid–solid transition in the blend with PF5-Y5, resulting in a polymer blend with a reduced level of donor aggregation.

When 2% CN is introduced into the blend solution, the drying process is slowed down, and an extended liquid–solid transition stage can be observed, evidenced by the continuous spectral shifts and PL quenching of both PBDB-T and PF5-Y5 after **t2** (Figure 1b,d). It significantly prolongs the overall liquid–solid transition (from **t1** to **t3**) during the blend drying. This is expected considering the higher boiling point of CN (263 °C) compared to that of the host solvent CB (131 °C). Apart from the prolonged drying time, the most iconic feature is that the red-shift of the PBDB-T emission maximum appears during the liquid–solid transition. It shows a rapid red-shift from 670 to 675 nm in the first liquid–solid transition (**t1** < **t** < **t2**), following a further red-shift in the second liquid–solid transition (**t2** < **t** < **t3**), reaching about 678 nm. A similar spectral dynamics of PBDB-T can also be observed in the corresponding in situ absorption measurement (Figure S4b). The overall spectral shifts of the PF5-Y5 emission peak in the blend with CN, however, are quite similar to that in the blend without CN. The emission peak of PF5-Y5 at 830 nm at **t0** in both blends shifts to 850 nm in the dried films.

The in situ PL and absorbance measurements are further conducted in the blends with different volume percents of CN (from 1 v/v% to 8 v/v%); the results show that, although the blends with increasing CN load exhibit longer second liquid–solid transition stages (from **t2** to **t3**, Figure S5), the size of spectral shifts of PBDB-T and PF5-Y5 emission peaks in the blend films are independent of the amount of CN (Figure S6). Analyzing the observed different drying dynamics, we conclude that the addition of CN in the BHJ slows down the drying and promotes the aggregation of PBDB-T during the liquid–solid transition, and as a result, an increased aggregation of PBDB-T could be expected in the resulting BHJ films, as observed for the pure PBDB-T films and PBDB-T:N2200 blends.^30^

As for the LS intensities (Figure 1e,f), both blends show abrupt LS enhancement from **t1** to **t2**, and the blend with CN exhibits extra LS fluctuation from **t2** to **t3**. Overall, during the complete film drying, the blend with CN features larger LS increases than the film without CN. Based on our previous study,^30^ the more pronounced LS increase in Figure 1f might indicate CN results in a rougher surface, which might be resulting in the enlargement in domain sizes of the corresponding blend films.

To verify the effect of CN on the film roughness, the surfaces of the dried films without and with 2% CN are imaged by atomic force microscopy (AFM). As shown in Figure 2a,b, both blend films show relatively flat surfaces with a slightly coarser nanostructure for the film with CN, as indicated by the root-mean-square (RMS) roughness of 2.0 nm, compared to the one of the film cast from neat CB (RMS roughness = 1.4 nm). It is indeed not unexpected that the slower-drying all-polymer blend film results in more developed nanostructures. Figure 2c presents the intensity profiles along the in-plane direction, resulting from grazing-incidence small-angle X-ray scattering (GISAXS) measurements, shown in Figure S7, fitted with the Debye–Anderson–Brumberger (DAB) model and the fractal-like network model.^31^ An average in-plane domain size in the blend films with CN and without CN is calculated to be ∼29 and ∼24 nm, respectively, matching well the observed trends of LS intensity changes during drying as well as the morphologies and trend in roughness obtained from AFM. We propose that the increased surface roughness and domain sizes in the film coated from CB with 2% CN are due to the enhanced PBDB-T aggregation, indicated by the PL spectra.

**Figure 2:**
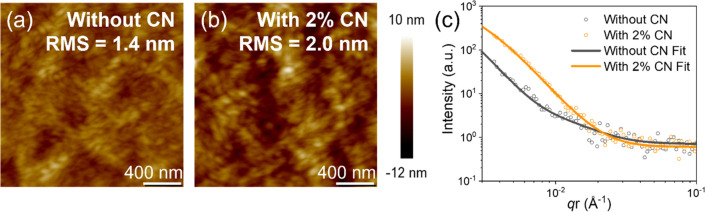
AFM height images of blade-coated PBDB-T:PF5-Y5 films from CB solutions (a) without and (b) with 2% CN. (c) GISAXS intensity profile of blade-coated PBDB-T:PF5-Y5 films without and with 2% CN.

Next, the polymer packing behavior and orientation are further probed by grazing incidence wide-angle X-ray scattering (GIWAXS). Figures 3 and S8 show the two-dimensional (2D) GIWAXS patterns of blade-coated PBDB-T:PF5-Y5 without and with 2% CN as well as neat PBDB-T and PF5-Y5 films, respectively. The corresponding linecuts along the in-plane (IP) and out-of-plane (OOP) direction for blend and pristine samples are also shown in Figure 3c,d. PBDB-T exhibits a bimodal lamellar peak at *q*_*z*_ ≈ 0.315 Å^–1^ (*d* = 19.9 Å) and *q*_r_ ≈ 0.300 Å^–1^ (*d* = 20.9 Å), while PF5-Y5 shows a lamellar peak at *q*_r_ ≈ 0.330 Å^–1^ (*d* = 19.0 Å). Both blend samples show lamella peaks at *q*_*z*_ ≈ 0.315 Å^–1^ (*d* = 19.9 Å) and *q*_r_ ≈ 0.300 Å^–1^ (*d* = 20.9 Å) and the π–π stacking peak at *q*_*z*_ = 1.70 Å^–1^ (*d* = 3.70 Å) (Figure 4c). The addition of CN in the BHJ leads to a significant intensity enhancement in the (100) lamellar peak in the IP direction and a slightly increased (010) π–π peak at 1.70 Å^–1^ in the OOP direction (Figure 3d). The crystal coherence length (CCL) of the (100) peak in the IP is calculated to be 4.03 and 7.44 nm for the blends without and with CN, respectively, and the CCL of the (010) peak in the OOP direction is 1.19 nm for the as-cast blend and 1.28 nm for the blend with CN. The enhanced peak intensities and CCLs clearly demonstrate that the addition of CN leads to an enhancement of the crystallinity within the film, which agrees well with the in situ PL results.

**Figure 3:**
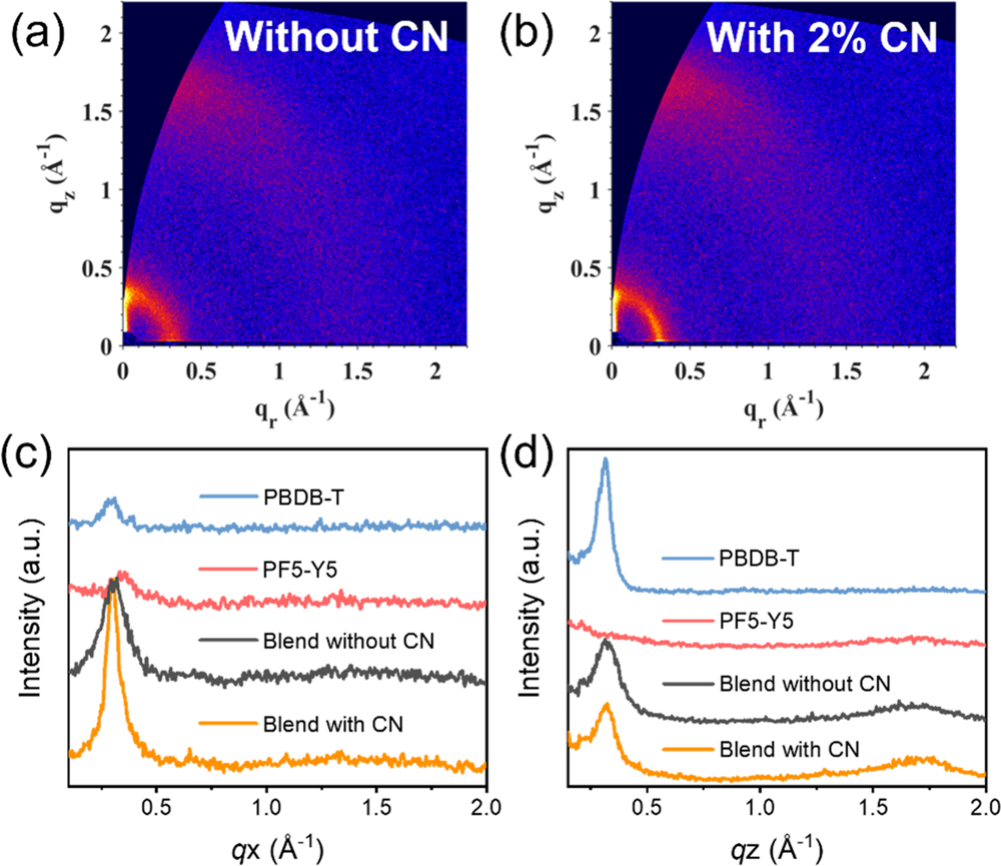
2D GIWAXS pattern of PBDB-T:PF5-Y5 blends films (a) without CN and (b) with 2% CN, and the corresponding intensity profiles of pristine and blend films along (c) in-plane and (d) out-of-plane directions.

**Figure 4:**
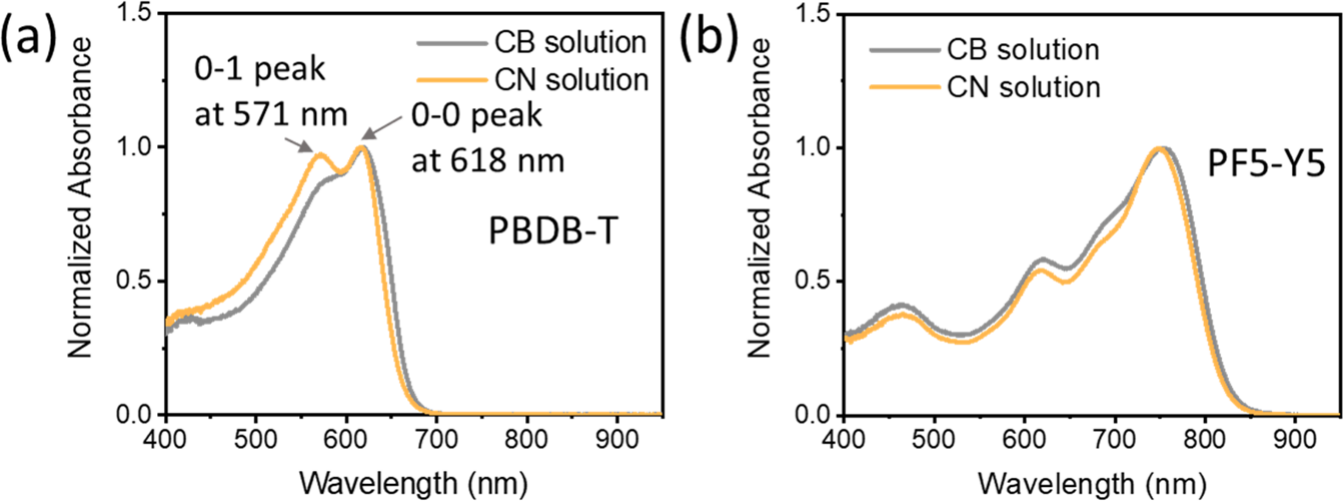
Absorption spectra of (a) PBDB-T and (b) PF5-Y5 solutions using CB and CN as the solvent.

From the studies above, we have concluded that a PBDB-T:PF5-Y5 blend film coated from CB is relatively free from PBDB-T aggregation. Contrary to this, the addition of 2% or more CN selectively promotes the aggregation of PBDB-T, leading to a blend film with larger domains and enhanced crystallinity. To explain the selectivity of CN in the PBDB-T:PF5-Y5 blend, we first check the possible solubility difference of PBDB-T in CB and CN, by determining its Hansen solubility parameters (HSP) through performing a series of solubility tests in 32 solvents, as described earlier.^32^ The resulting Hansen solubility sphere for PBDB-T, obtained from the HSPiP program, is shown in Figure S9, and the extracted values for the HSP parameters are given in Table S1. The distance between the center of the PBDB-T sphere and the points representing the solvents, *R*_a_, is a measure of solubility. The relative energy distance (RED) value, i.e., the ratio between *R*_a_ and the sphere radius *R*_0_, for PBDB-T and the two solvents, CB and CN, is 0.914 and 0.980, respectively, showing a slightly better solubility of PBDB-T in CB than in CN. This is in line with a promoted aggregation of the donor polymer in the presence of CN, as described above. As CN has a lower vapor pressure and evaporates slower than CB, the aggregation is strengthened.

Next, both polymers are separately dissolved in pure CB and pure CN with a concentration of 0.1 g/L, and their absorption spectra are recorded in solution. As shown in Figure 4a, the absorption spectra of PBDB-T in CB and in CN exhibit two vibronic peaks corresponding with the 0–0 transition located around 618 nm and the 0–1 transition around 571 nm, whereas the absorption spectra of PF5-Y5 in both solvents, shown in Figure 4b, are rather similar. The vibronic peaks are indicative of aggregation, and their intensity ratio *I*_0–0_/*I*_0–1_ is a measure of the degree of aggregation.^33,34^ The ratio *I*_0–0_/*I*_0–1_ is 1.16 for PBDB-T solutions in CB and 1.04 in CN. The lower *I*_0–0_/*I*_0–1_ ratio of PBDB-T in CN indicates that PBDB-T has a lower degree of preaggregation in CN than in CB.^15,35^ However, in the PBDB-T:PF5-Y5 binary blend solution, the aggregation behavior of PBDB-T is different. More specifically, PBDB-T shows less preaggregation when it is dissolved in CN, whereas in the PBDB-T:PF5-Y5 binary blend solution with CN as the additive, PBDB-T self-aggregation is somehow promoted. The role of adding CN in our all-polymer system could hence be to make the PBDB-T chains less available for interaction with PF5-Y5 during the early stage of the film-drying process, thus promoting self-aggregation of PBDB-T in the mixed solvent system. In another word, the presence of CN shifts the balance of polymer interactions in the binary blend solution, from a possibly strong PBDB-T/PF5-Y5 interaction to a more favorable PBDB-T self-aggregation. As a result, PBDB-T in the CN phase is able to form PBDB-T aggregates during the slow solvent evaporation, which is revealed by the red-shifted PBDB-T emission in Figure 1b, and results in enlarged pure domains and increased crystallinity of the corresponding blend film, while in the absence of CN, the blend film dries faster and the self-aggregation of PBDB-T is hindered.

Next, the measurement of electron and hole mobilities is carried out to evaluate the possible contributions from CN to the charge transport properties in the device. The charge carrier mobilities are calculated from the space-charge-limited current (SCLC) region in the current density–voltage (*J–V*) curves that are recorded from the single carrier devices in dark (Figure 5). As shown in Table 1, compared to the blend without CN, the blend with 2% CN displays more than 4 times increase in hole mobility (μ_h_); on the contrary, no obvious changes can be found in their electron mobilities (μ_e_). It has been widely reported that the BHJ blends with a higher degree of crystallinity usually result in improved charge transport properties. Therefore, the enhanced hole mobility in our study clearly indicates that the high-crystallinity phases mainly exist in PBDB-T. Besides, thanks to the enhanced hole mobility, more balanced charge transport μ_e_/μ_h_ is achieved in all-PSCs with CN, which contributes to the increased FF in the corresponding devices.

**Figure 5:**
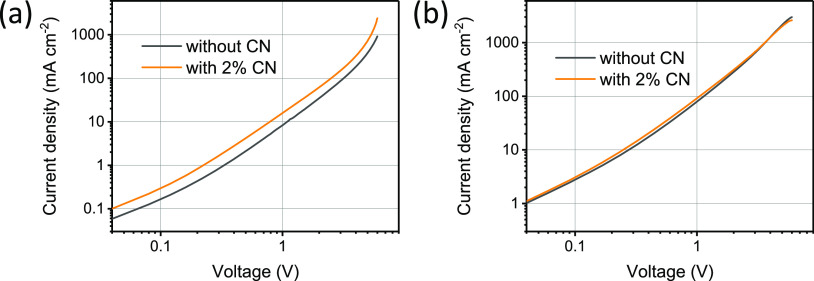
Dark *J–V* curves of (a) hole-only and (b) electron-only devices based on PBDB-T:PF5-Y5 blends.

**Table 1:** Charge Carrier Mobilities of the PBDB-T:PF5-Y5 Blend without or with 2% CN

	hole mobility (cm^2^ V^–1^ s^–1^)	electron mobility (cm^2^ V^–1^ s^–1^)	μ_e_/μ_h_
without CN	3.04 × 10^–5^	1.23 × 10^–4^	4.04
with 2% CN	1.30 × 10^–4^	1.51 × 10^–4^	1.16

The enhancement of FF and PCE (from 12.41% to 13.61%) in devices processed with 2% CN is not only demonstrated in the previous publication^7^ but also in the all-PSCs fabricated by both blade coating and spin coating in our lab. The characterization details of our devices are shown in Table S2 and Figure S10. Although the overall performance of our device is lower than the reported values, the effect of CN on the device performance is consistent: compared to the reference devices, which are processed in CB without CN, the devices with CN maintain a constant short-circuit current (*J*_SC_), with a slightly improved *V*_OC_, an obviously increased FF, and, eventually, an enhanced PCE. A voltage loss analysis is carried out to explore the reason for the improvement of *V*_OC_. Details of calculation are provided in Supplementary Note 1 in the Supporting Information. Results show that the device with 2% CN achieves an ca. 15 mV lower nonradiative energy loss than the reference device, indicating that the crystallized PBDB-T segments might also help to block the nonradiative decay channels of excitons, resulting in a slightly higher *V*_OC_ in the device with CN.

In this study, the influence of the solvent additive, in particular, the addition of CN, in the highly efficient PBDB-T:PF5-Y5 all-polymer blend has been analyzed and understood with the aid of in situ optical spectroscopies. The aggregation of donor and acceptor during the film drying could be tracked separately during film formation, thanks to their well-distinguished absorption and emission spectra. Interestingly, it was found that only the self-aggregation of PBDB-T is promoted by 2% CN to the CB. Spectroscopy studies in polymer solutions indicate that PBDB-T chains in pure CN solution are in fact less aggregated, making the PBDB-T chains less available for interaction with PF5-Y5 during the early stage of the film-drying process, thus promoting self-aggregation of PBDB-T in the mixed solvent system and forming larger donor phases with a higher degree of crystallinity in the dry film. As a result, the hole mobility in the blend film prepared with CN is increased, whereas the electron mobility is not affected by CN. Overall, the improved device performance after introducing CN is mainly due to the improved FF, because of balanced charge carrier mobilities, in addition to a slight improvement in *V*_OC_, because of reduced energy loss by suppressed nonradiative recombination. Thus, our study provides an in-depth perspective on the role of CN as a solvent additive in a new all-polymer BHJ system, which is valuable for the further development of highly efficient all-PSCs.

## Supporting Information Available

Experimental details. Complete evolutions of PL and absorption spectra during blend film formation, GISAXS and GIWAXS patterns of blend and pristine films, PL and absorption spectra of pristine polymer films, solubility sphere for PBDB-T in Hansen space, and device performance with voltage losses calculation (PDF)
Transparent Peer Review report available (PDF)
