# Supplementary material for: In Situ Optical Spectroscopy Demonstrates the Effect of Solvent Additive in the Formation of All-Polymer Solar Cells
Source: J Phys Chem Lett. 2022 Dec 13;13(50):11696–702. doi: 10.1021/acs.jpclett.2c03397 (PMC9791685; doi:10.1021/acs.jpclett.2c03397)
Supplement: Supplementary file 1 — jz2c03397_si_001.pdf [file jz2c03397_si_001.pdf]

Supporting Information

In Situ Optical Spectroscopy Demonstrates the Effect of Solvent Additive in the Formation of All-Polymer Solar Cells

Yanfeng Liu,[a, b] *Qunping Fan,*[c, d] *Heng Liu,*[e] *Ishita Jalan,*[f] *Yingzhi Jin,*[g] *Jan van Stam,*[f, *] *Ellen Moons,*[h] *Ergang Wang,*[c, *] *Xinhui Lu,*[e, *] *Olle Inganäs,*[a] *Fengling Zhang* [a, *]

[a] Biomolecular and Organic Electronics, Department of Physics, Chemistry and Biology, Linköping University. Linköping SE-581 83, Sweden.

[b] College of Materials and Textile Engineering, Nanotechnology Research Institute, Jiaxing University, Jiaxing 314001, China.

[c] Department of Chemistry and Chemical Engineering, Chalmers University of Technology, Göteborg SE-412 96, Sweden.

[d] State Key Laboratory for Mechanical Behavior of Materials, Xi'an Jiaotong University, Xi'an 710049, China.

[e] Department of Physics, The Chinese University of Hong Kong. Shatin 999077, Hong Kong, China.

[f] Department of Engineering and Chemical Sciences, Karlstad University, SE-651 88, Karlstad, Sweden.

[g] China-Australia Institute for Advanced Materials and Manufacturing, Jiaxing University, Jiaxing 314001, China.

[h] Department of Engineering and Physics, Karlstad University, SE-651 88, Karlstad, Sweden.

Experimental Methods

***Materials*:** PBDB-T (Mw = 45 kDa, PDI = 2.0), PDINO, and PEI were purchased from Organtec Ltd. MoO_3, CB, and CN were purchased from Sigma-Aldrich. PEDOT:PSS (AI 4083) was purchased from Heraeus. All chemicals were used as received. ITO-patterned glass substrates were purchased from CSG Holding Co., Ltd. PF5-Y5 (Mw = 25 kDa, PDI = 2.0) was synthesized according to the previously reported procedure.[1]

***In-situ PL and absorption*:** The *in-situ* PL setup was reported in our previous article.[2] The all-polymer solutions were prepared by mixing PBDB-T and PF5-Y5 with a weight ratio of 1:0.75, then dissolving the polymer blend in CB with a total concentration of 17 mg/mL. For solutions with CN as the additive, different amount of CN was added into the polymer solutions with volume percent from 1 % to 8 %. The polymer solutions were printed using a blade coater (Erichsen 510), the coating conditions were the same as the blade-coated device fabrication. The polymer films were excited by a 532 nm laser diode, the emission spectra and the scattered laser signal were continuously recorded by the spectrometer (QE-Pro, Ocean Optics). For the *in-situ* absorption measurement, the setup was slightly modified to create extra space underneath the substrate for another optical fiber, in order to probe the evolution of absorption by recording the transmittance spectra.

***Absorption of polymer solutions*:** The concentration of polymer solutions was kept as 0.1 mg/mL in quartz cuvettes for this measurement. The cuvette then was placed in the cuvette holder (CVH100, ThroLabs) with two optical fibers connected on each side, one is used to guide white light from a tungsten lamp (DH 2000, Ocean Optics); the other one is to collect transmitted light to the spectrometer (QE-Pro).

GIWAXS and GISAXS measurements: GIWAXS characterization of the pristine and blend films was performed with a Xeuss 2.0 SAXS/WAXS laboratory beamline using a Cu X-ray source (8.05 keV, 1.54 Å) and a Pilatus3R 300K detector. The incidence angle is 0.2 °. Both GIWAXS and GISAXS samples are prepared on silicon substrates.

AFM measurement: AFM images were obtained by a Dimension 3100 (Veeco Digital Instruments) in air. The tapping mode with an Al-coated PPP-NCHR-20 silicon AFM tip (NANOSENSORS) was employed during the measurement.

***Solubility test and HSP calculations*:** In short, PBDB-T was first dissolved in 32 different solvents in a series of concentrations, the solubility of PBDB-T in these solvents was scored, the scores were then imported into the HSPiP software, and the HSP values of PBDB-T could be calculated. The calculation details can be found in both SI and previous publication.[3]

Fabrication and characterization of the single-carrier devices: The hole- and electron-only devices were fabricated using the structures of ITO/PEDOT:PSS/active layer/MoO_3/Ag and ITO/PEI/active layer/PDINO/Al, respectively. The preparation of the active layer was the same as the preparation of the solar cells. For the hole-only device, 10 nm MoO_3 and 100 nm silver were thermally evaporated under vacuum. For the electron-only device, the PEI iso-propanol solution (0.5 mg/mL) was spin-coated on the ITO substrate to form the hole-blocking layer. The *J-V* curves of the single-carrier devices were recorded by Keithley 2400 source meter in the dark. The hole and electron mobilities were calculated by selectively fitting the SCLC region in the *J-V* curves, following the protocol of a previously published article.[4]

Fabrication and characterization of OSCs: All solar cells were fabricated using a structure of ITO/PEDOT:PSS/active layer/PDINO/Al. A 40 nm thick PEDOT:PSS was spin-coated on an ITO-

patterned glass substrate and thermally annealed at 150 °C for 15 min. For spin-coated devices, a 100 nm active layer (PBDB-T:PF5-Y5, weight ratio 1:0.75, with a total concentration of 17 mg/mL in chlorobenzene) was spin-coated on top of the PEDOT:PSS layer in the N_2 filled glove box at room temperature, following thermal annealing at 100 °C for 10 min. For the blade-coated device, the same active layer solution was coated on top of the PEDOT:PSS layer in air at room temperature, and following the same thermal annealing process. The thickness of the blade-coated active layer was kept the same as that in spin-coated devices (around 100 nm). The PDINO solution (1 mg/mL in methanol) was spin-coated on top of the active layer to form the electron transport layer. The device was completed by thermally evaporating 100 nm aluminum under vacuum (1.0 $\times 10^{-6}$ mbar) as the top electrode. The active area was defined to be 0.047 cm^{-2} by using a shadow mask.

The *J-V* curves of the unencapsulated devices were measured under 1 sun (100 mW cm^{-2}, AM 1.5G) in air with an LSH-7320 solar simulator and a Keithley 2400 source meter. The external quantum efficiency (EQE) spectra were measured using a QE-R3011 system from Enli Technology Co., Ltd.

The electroluminescence quantum efficiency (EQE_{EL}) of the device was obtained from a home-made detection system, including a Hamamatsu silicon photodiode 1010B, a Keithley 2400 source meter, and a Keithley 485 picoammeter. The high-sensitive EQE was measured by the Fourier transform photocurrent spectroscopy (FTPS), which was conducted in a home-made system based on Vertex 70 Fourier transform infrared spectroscopy from Bruker Optics.

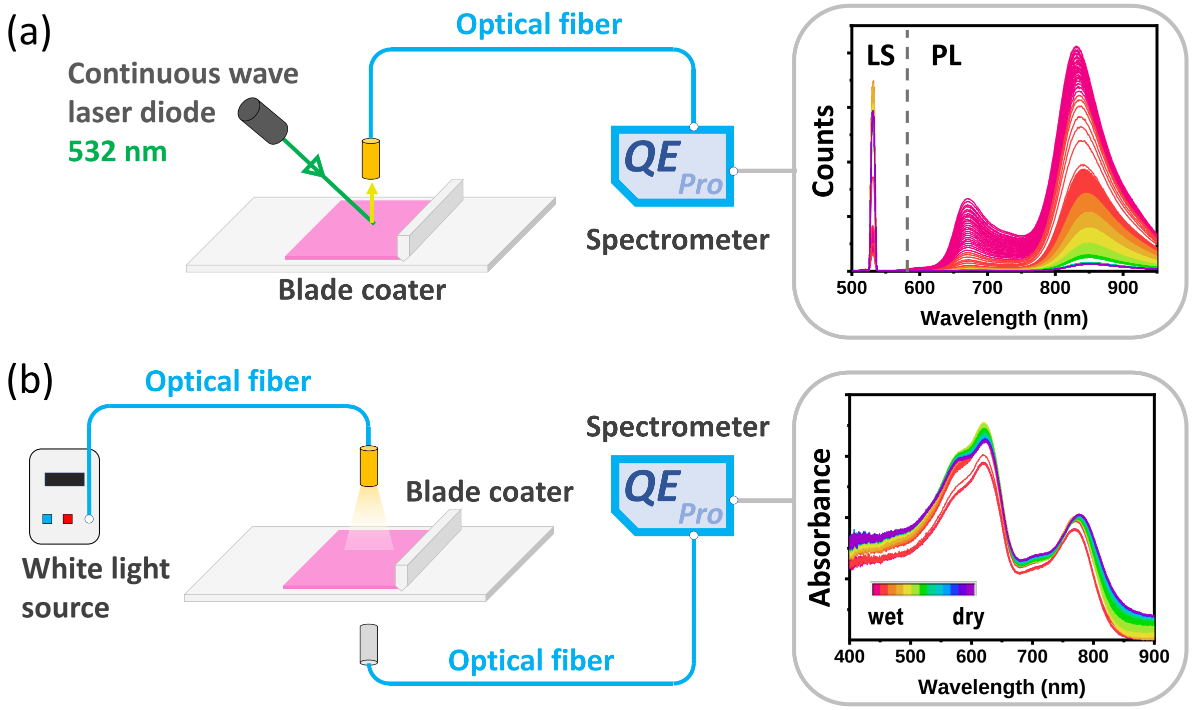

Figure S1. Schematic diagrams of the *in-situ* setup for measuring (a) PL emission and LS signal from the studied blend captured simultaneously with its PL emission; (b) absorption during BHJ formation.

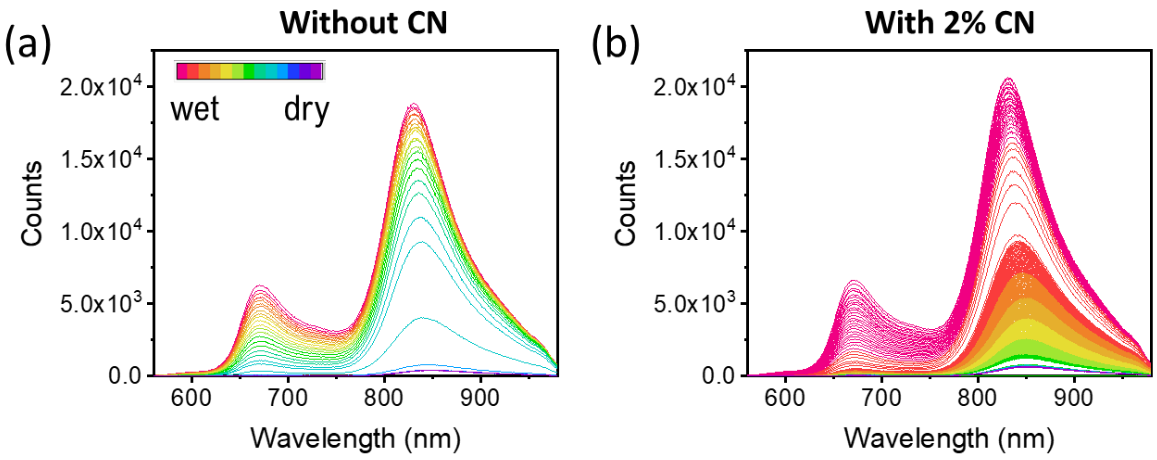

Figure S2. Complete PL spectra evolution during the drying process of blade-coated PBDB-T:PF5-Y5 blend (a) without or (b) with 2% CN.

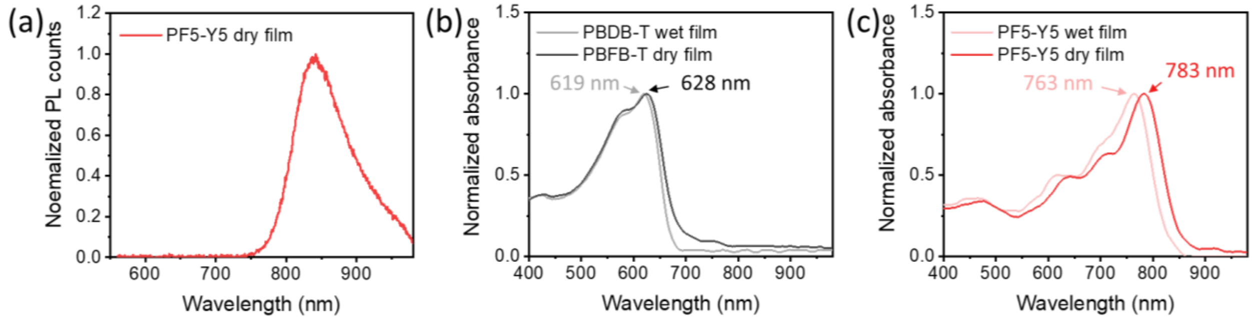

Figure S3. (a) PL emission spectrum of pristine PF5-Y5 dry film. (b) Absorption spectra of pristine PBDB-T and (c) PF5-Y5 in wet and dry film during *in-situ* absorption measurement.

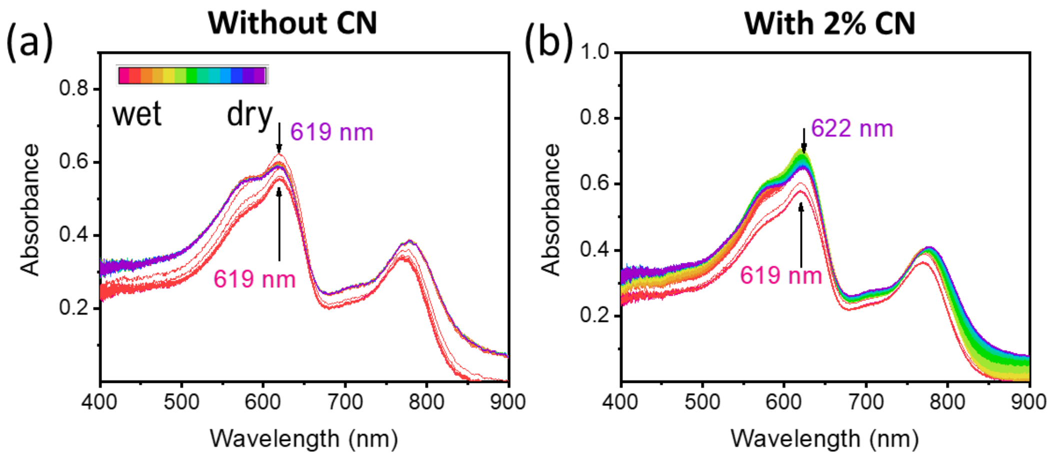

Figure S4. Complete absorption spectra evolution during the drying process of blade-coated PBDB-T:PF5-Y5 blend (a) without or (b) with 2% CN. The PBDB-T absorption peak positions in wet blend films (pink) and dry blend films (purple) are marked in the plots.

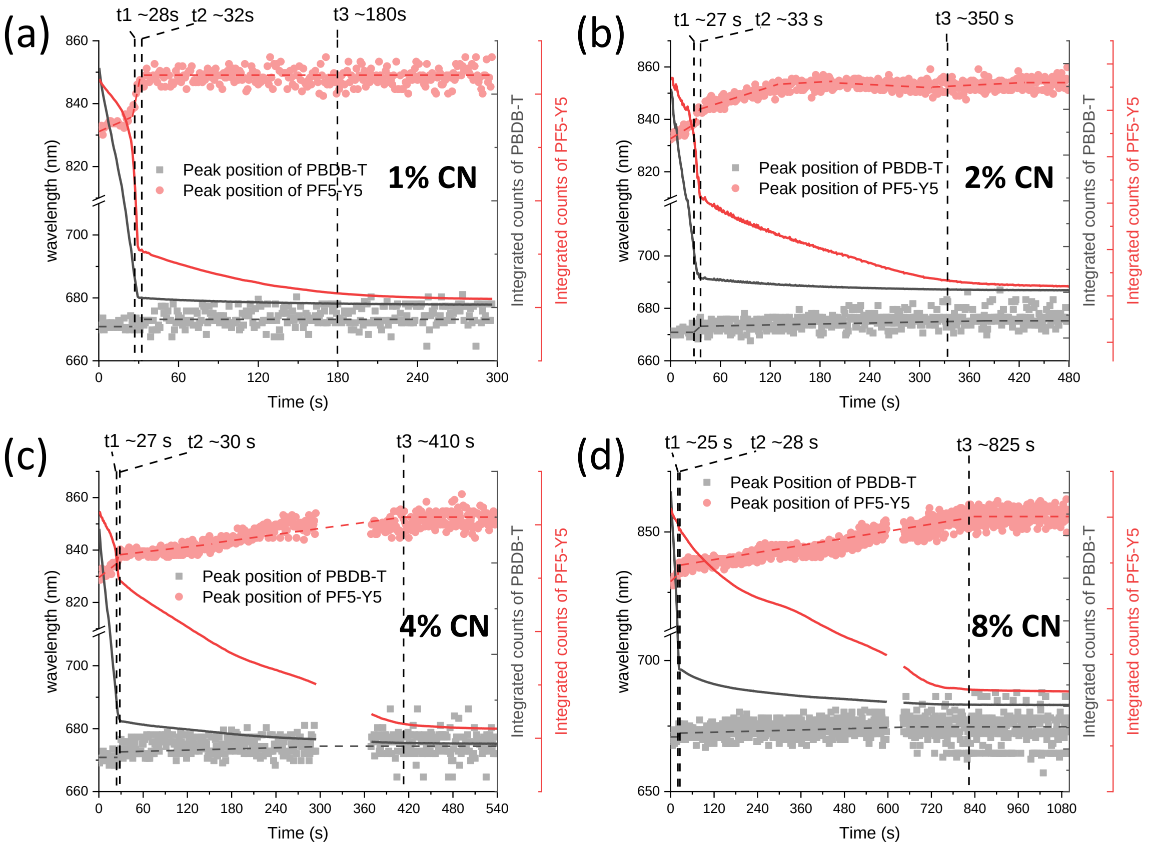

Figure S5. The evolution of peak position and peak intensity of PBDB-T and PF5-Y5 in the blend, with (a) 1%, (b) 2%, (c) 4%, and (d) 8% of CN. The value of t1, t2, and t3 for each drying condition are marked in the plots. Dash lines are guides to the eye.

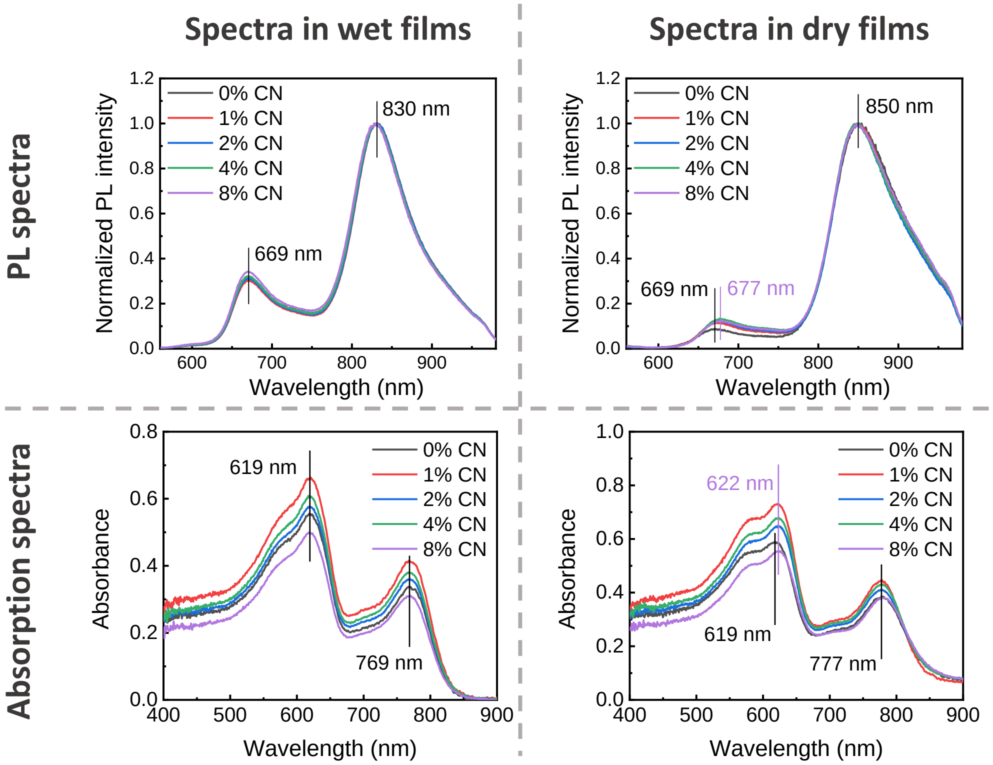

Figure S6. PL and absorption spectra of blends with different amount of CN in wet (t0) and in dry (after t3) conditions.

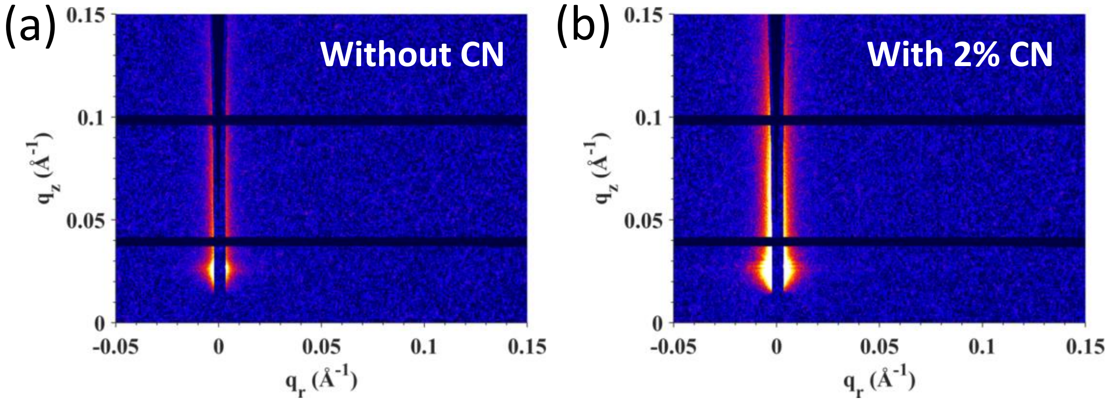

Figure S7. 2D GISAXS pattern of blade-coated PBDB-T:PF5-Y5 (a) without CN and (b) with 2% CN.

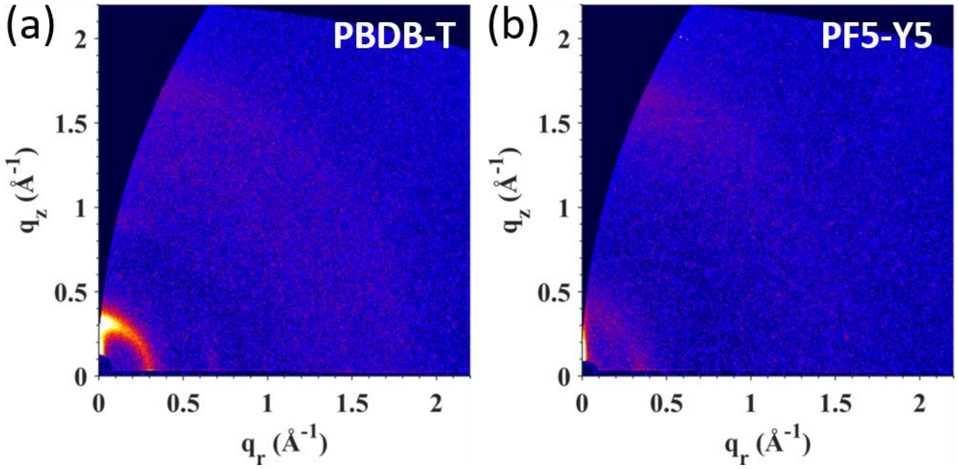

Figure S8. 2D GIWAXS pattern of blade-coated pristine (a) PBDB-T and (b) PF5-Y5.

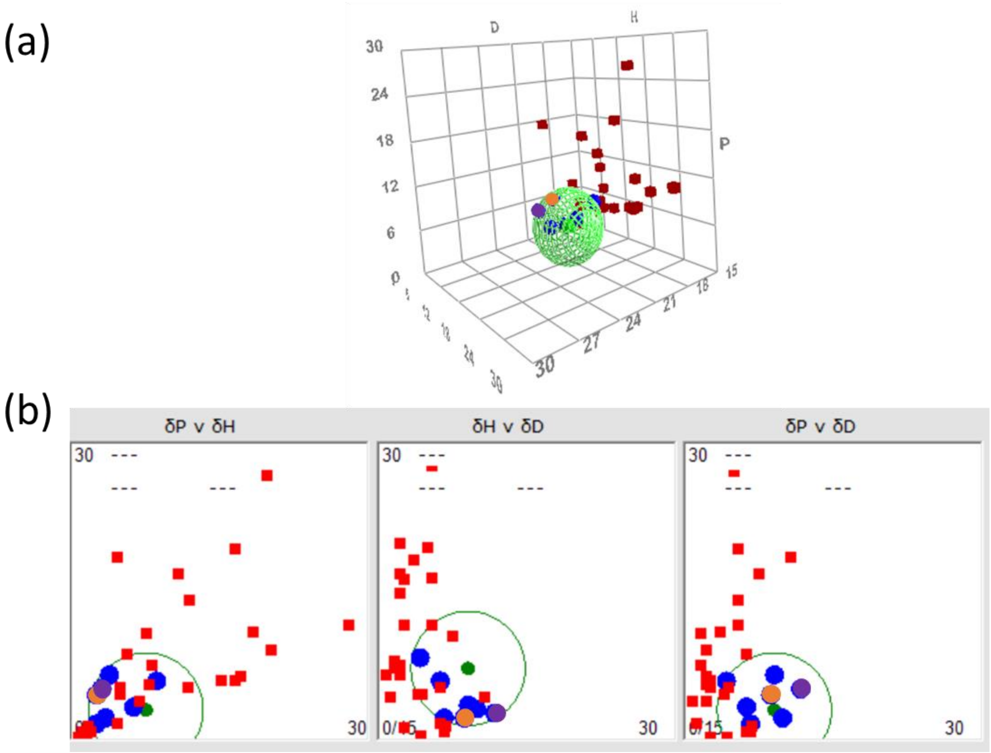

Figure S9. (a) The solubility sphere for PBDB-T in three-dimensional (3-D) Hansen space, separates bad solvents (outside of the sphere) from good solvents (inside of the sphere), and is the results of solubility tests of PBDB-T in a set of solvents, processed by the HSPiP program. The

coordinates of the center of the green sphere represent the three HSP parameters of PBDB-T, for the dispersion interaction (δD, or axis D), polar interaction (δP, or axis P), and hydrogen bonding interaction (δH, or axis H). The HSP values of CB (orange dot) and CN (purple dot) are also plotted. (b) The relative position between PBDB-T (green dot) and the solvents can be read more easily in the two-dimensional projections of the Hansen sphere.

Table S1. The HSP values of PBDB-T, CB, and CN in the Hansen space. R_0 is the radius of the sphere (the green sphere in Figure S8) in Hansen space for PBDB-T solutions of 1 mg/ml and 10 mg/mL. The distance between the center of the PBDB-T sphere and the points representing the solvents, R_a, is a measure for the solubility. R_a can be calculated by the following equation:

$$R_a^2 = 4(\delta D_2 - \delta D_1)^2 + (\delta P_2 - \delta P_1)^2 + (\delta H_2 - \delta H_1)^2$$

The relative energy distance (RED) value is the ratio of R_a/R_0. A RED value smaller than 1 indicates that PBDB-T dissolves well in a given solvent. Here the RED values for polymer PBDB-T and the two solvents (CB and CN) are similar, meaning PBDB-T should have a similar solubility when dissolved in CB and CN.

	δD	δP	δH	R_0	RED
PBDB-T	18.9	2.5	7.0	5.4	
CB	19.0	4.3	2.0		0.914
CN	20.5	4.9	2.5		0.980

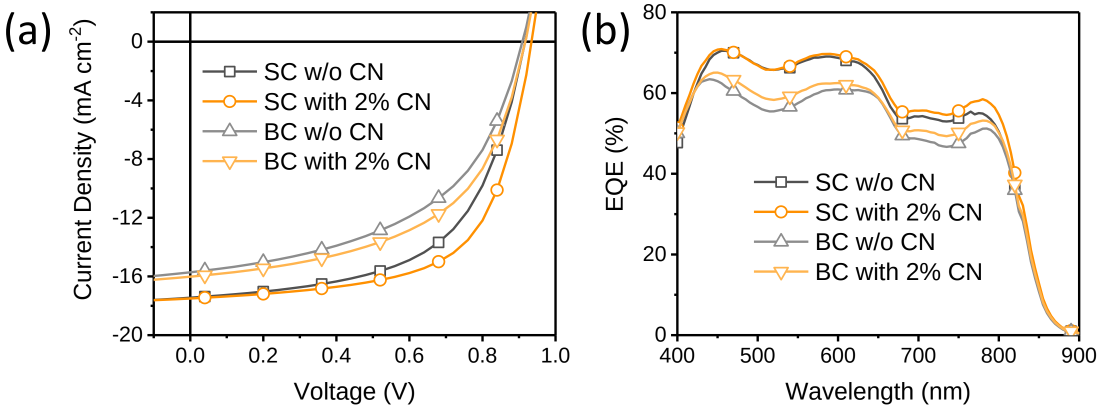

Figure S10. (a) The *J-V* and (b) EQE curves of spin-coated (SC) and blade-coated (BC) all-polymer solar cells based on PBDB-T:PF5-Y5 (1:0.75) without and with 2% CN. Spin-coated active layers are fabricated in an N_2-filled glove box, blade-coated active layers are fabricated in ambient air.

Table S2. Corresponding photovoltaic parameters of all-polymer solar cells based on PBDB-T:PF5-Y5 (1:0.75) without or with 2% CN. The average values with standard deviation are obtained from 20 devices.

	V_{OC} (V)	J_{SC} (mA cm^{-2})	FF (%)	PCE (%)	Integrated J_{SC} (mA cm^{-2})
SC w/o CN	0.917 (0.916±0.002)	17.45 (17.27±0.29)	58.2 (57.3±1.2)	9.30 (9.06±0.16)	17.13
SC 2% CN	0.934 (0.934±0.002)	17.50 (17.07±0.45)	63.4 (63.9±1.0)	10.37 (10.15±0.18)	17.27
BC w/o CN	0.909 (0.908±0.003)	15.72 (15.46±0.29)	50.8 (49.3±3.8)	7.26 (7.08±0.13)	15.49
BC 2% CN	0.917 (0.917±0.04)	16.02 (15.57±0.37)	54.3 (54.5±2.9)	7.98 (7.71±0.43)	15.71

Supplementary Note 1: Voltage losses analysis

The voltage loss analysis is conducted on the spin-coated solar cells to explore the reason for the V_{oc} improvement after adding CN. The calculations are based on the Shockley – Queisser (S-Q) limit theory, and results are summarized in Figure S10 and Table S3. In general, the total voltage loss V_{loss} in OSCs can be divided into three parts,

$$V_{loss} = \frac{E_g}{q} - V_{\mathrm{OC}} = \left(\frac{E_g}{q} - V_{\mathrm{OC}}^{\mathrm{SQ}}\right) + \left(V_{\mathrm{OC}}^{\mathrm{SQ}} - V_{\mathrm{OC}}^{\mathrm{rad}}\right) + \left(V_{\mathrm{OC}}^{\mathrm{rad}} - V_{\mathrm{OC}}\right) = \Delta V_1 + \Delta V_2 + \Delta V_3$$

where E_g is the optical bandgap of the active layer, which is defined by the 1st derivative of the high-sensitive EQE curves (Figure S11), $V_{\mathrm{OC}}^{\mathrm{SQ}}$ is the maximum V_{OC} under S-Q limit, and $V_{\mathrm{OC}}^{\mathrm{rad}}$ is the radiative limit V_{OC}. ΔV_1 is the loss from radiative recombination, it only depends on the bandgap and is inevitable in any kind of solar cells. ΔV_2 is due to the mismatch of EQE between the real solar cell and the ideal one, which also commonly exists in OSCs. Both ΔV_1 and ΔV_2 are similar for the device with or without CN, whereas the only difference is found in the loss from the non-radiative recombination ($\Delta V_{3,\mathrm{Exp}}$, calculated from the $\mathrm{EQE_{EL}}$ results by using the equation $\Delta V_{3,\mathrm{Exp}} = -\frac{kT}{q}\ln(EQE_{\mathrm{EL}})$). The device with 2% CN shows a ca. 15 mV lower ΔV_3 than device without CN, indicating that the crystalized PBDB-T segments might also help to block the nonradiative decay channels of excitons, thus promote more efficient charge separation at the donor/acceptor interface, resulting a slightly higher V_{OC} in the device with CN.

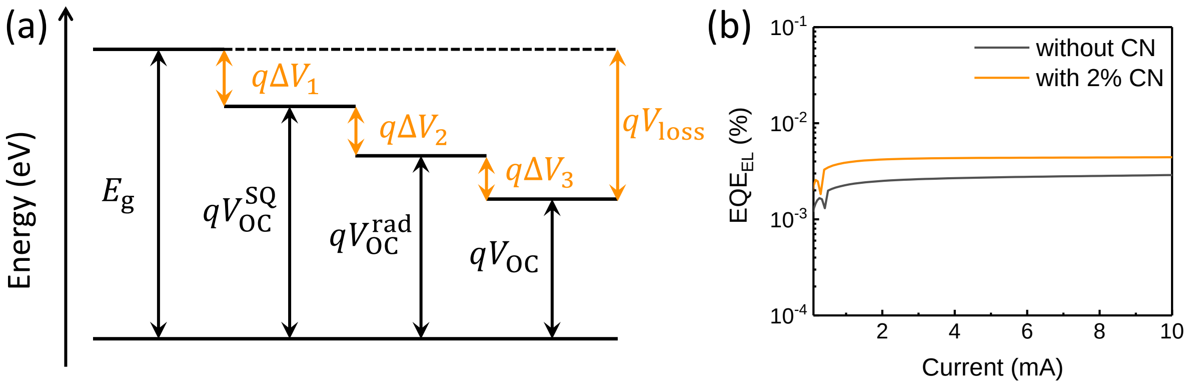

Figure S11. (a) Energy diagram of the voltage losses in OSCs. (b) EQE_{EL} of all-PSCs based on PBDB-T:PF5-Y5.

Table S3. The voltage loss calculation of all-PSCs based on PBDB-T:PF5-Y5 by using S-Q limit theory.

	E_g (eV)	qV_{OC} (V)	E_{loss} (eV)	qV_{OC}^{SQ} (V)	qV_{OC}^{rad} (V)	ΔV_1 (V)	ΔV_2 (V)	ΔV_3 (V)	EQE_{EL} (%)	Exp. ΔV_3^{Exp} (V)
Without CN	1.503	0.917	0.586	1.229	1.189	0.274	0.040	0.272	0.002	0.278
With 2% CN	1.503	0.934	0.569	1.229	1.186	0.274	0.043	0.252	0.004	0.263

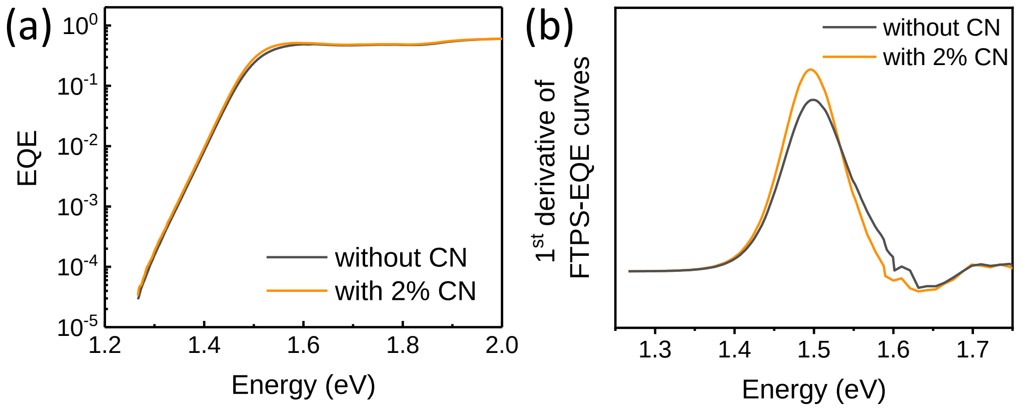

Figure S12. (a) High-sensitive EQE measurements of PBDB-T:PF5-Y5 without CN and with 2% CN. (b) the corresponding derivatives of the EQE curves.

REFERENCES

(1) Fan, Q.; An, Q.; Lin, Y.; Xia, Y.; Li, Q.; Zhang, M.; Su, W.; Peng, W.; Zhang, C.; Liu, F.; et al. Over 14% Efficiency All-Polymer Solar Cells Enabled by a Low Bandgap Polymer Acceptor with Low Energy Loss and Efficient Charge Separation. *Energy Environ. Sci.* **2020**, *13*, 5017-5027.
(2) Liu, Y.; Yangui, A.; Zhang, R.; Kiligaridis, A.; Moons, E.; Gao, F.; Inganäs, O.; Scheblykin, I. G.; Zhang, F. In Situ Optical Studies on Morphology Formation in Organic Photovoltaic Blends. *Small Methods* **2021**, *5*, 2100585.
(3) Jalan, I.; Lundin, L.; van Stam, J. Using Solubility Parameters to Model More Environmentally Friendly Solvent Blends for Organic Solar Cell Active Layers. *Materials* **2019**, *12*, 3889.
(4) Felekidis, N.; Melianas, A.; Kemerink, M. Automated Open-Source Software for Charge Transport Analysis in Single-Carrier Organic Semiconductor Diodes. *Org. Electron.* **2018**, *61*, 318-328.
