# Supplementary material for: In Situ Optical Spectroscopy Demonstrates the Effect of Solvent Additive in the Formation of All-Polymer Solar Cells
Source: J Phys Chem Lett. 2022 Dec 13;13(50):11696–702. doi: 10.1021/acs.jpclett.2c03397 (PMC9791685; doi:10.1021/acs.jpclett.2c03397)
Supplement: Supplementary file 2 — jz2c03397_si_002.pdf [file jz2c03397_si_002.pdf]

jz-2022-03397f.R1

Name: Peer Review Information for "In-*Situ* Optical Spectroscopy Demonstrates the Effect of Solvent Additive in the Formation of All-Polymer Solar Cells"

First Round of Reviewer Comments

Reviewer: 1

Comments to the Author

Aggregation of donor and acceptor is crucial effect for high-performance organic solar cells. The manuscript by Liu et al used in-situ optical spectroscopy to study the different aggregation of polymer donor with/without CN additives. The results from the in-situ optical spectroscopy is well consistent with the conclusions from ex-situ methods, indicates the in-situ PL is a useful method to study the drying process of organic solar cells. Herein, the manuscript should be accepted after these minor problems:

1) When the authors described the drying times, only t1, t2, … was used. I think the authors should label the time in the Figures. For example, t1=21s in Figure 1a as they do in Figure S5.

2) The authors used RED value to prove the better slightly better solubility of PBDB-T in CB than in CN. While in the UV-Vis spectra, “the ratio I0-0/I0-1 is 1.16 for PBDB-T solutions in CB and 1.04 in CN. The lower I0-0/I0-1 ratio of PBDB-T in CN indicates that PBDB-T has a lower degree of pre-aggregation in CN than in CB.” The lower I0-0/I0-1 ratio of PBDB-T is always observed in temperature-dependent UV-Vis when the temperature increased. Thus, the lower I0-0/I0-1 ratio of PBDB-T in CN may also indicate the better solubility of PBDB-T in the CN. Therefore, it’s better to give or test the solubility of PBDB-T in CB and CN.

3) “However, in the PBDB-T:PF5-Y5 binary blend solution, the balance of interactions is different. In CB with added CN, the PBDB-T chains self-aggregate more than in CB.” More descriptions should be added to explain the balance of interactions.

Reviewer: 2

Comments to the Author

The manuscript by Liu et.al investigated the role of the comment additive CN in determining the photovoltaic performance of the all-polymer organic solar cells. The in-situ PL and absorption spectroscopy were used in this work and the results indicated that CN promoted self-aggregation of the donor PBDB-T during the drying process of the blend film. It resulted in the improved hole mobility and lower non-radiative recombination voltage loss and higher photovoltaic performance of the PBDB-T:PF5-Y5 organic solar cells. This manuscript deepened our understanding of the role of the solvent additive

CN in drying dynamics for all-polymer solar cells. Therefore, I recommend the publication of this manuscript. A few specific suggestions are provided as follows for improving the manuscript.

1. The volume or weight content of the CN in CB is suggested to be indicated in the manuscript.

2. The molecular weight of polymers can greatly affect the device performance of all-polymer solar cells. The molecular weight and PDI value are suggested to be provided in this manuscript.

3. In page 18, the authors draw a conclusion: "…the device with 2% CN achieves a ca. 15 mV lower non-radiative energy loss than the reference device, indicating that the crystalized PBDB-T segments might also help to block the nonradiative decay channels of excitons, thus promoting more efficient charge separation at the donor/acceptor interface, resulting in a slightly higher VOC in the device with CN."

More analysis is needed on the relationship between "block the nonradiative decay channels of excitons" and the "more efficient charge separation at the donor/acceptor interface".

Author's Response to Peer Review Comments:

Dear Prof. Editor,

Thank you very much for your kind consideration on our manuscript. We appreciate reviewers for their constructive comments and suggestions. The manuscript has been carefully revised according to reviewers' suggestions, all the changes are highlighted (blue) in the revised manuscript and revised supporting information. Detailed point-by-point responses (blue and red) are presented as following.

We hope that you will accept our revised work for published in The Journal of Physical Chemistry Letters.

Yours sincerely,

Fengling Zhang

Reviewer(s)' Comments to Author:

Reviewer: 1

Recommendation: This paper is probably publishable, but major revision is needed; I do not need to see future revisions.

Comments:
Aggregation of donor and acceptor is crucial effect for high-performance organic solar cells. The manuscript by Liu et al used in-situ optical spectroscopy to study the different aggregation of polymer donor with/without CN additives. The results from the in-situ optical spectroscopy is well consistent with the conclusions from ex-situ methods, indicates the in-situ PL is a useful method to study the drying process of organic solar cells. Herein, the manuscript should be accepted after these minor problems:

We thank the reviewer for this positive comment.

1. When the authors described the drying times, only t1, t2, . was used. I think the authors should label the time in the Figures. For example, t1=21s in Figure 1a as they do in Figure S5.

As the reviewer's suggestion, times have been added in Figure 1 in the revised manuscript.

2. The authors used RED value to prove the better slightly better solubility of PBDB-T in CB than in CN. While in the UV-Vis spectra, "the ratio I0-0/I0-1 is 1.16 for PBDB-T solutions in CB and 1.04 in CN. The lower I0-0/I0-1 ratio of PBDB-T in CN indicates that PBDB-T has a lower degree of pre-aggregation in CN than in CB." The lower I0-0/I0-1 ratio of PBDB-T is always observed in temperature-dependent UV-Vis when the temperature increased. Thus, the lower I0-

0/I0-1 ratio of PBDB-T in CN may also indicate the better solubility of PBDB-T in the CN. Therefore, it's better to give or test the solubility of PBDB-T in CB and CN.

We thank the reviewer for the suggestion. The solubility of PBDB-T in both CB and CN was evaluated by determining its Hansen solubility parameters (HSP), and the corresponding results have been presented in the manuscript and supporting information. The experiment was conducted using the method described below: 32 solvents that cover the Hansen space were selected, PBDB-T was dissolved in each solvent at different concentrations, starting from a lower concentration (1 mg/mL) to a higher concentration (10 mg/mL). The dissolving state of PBDB-T in all the solutions was scored, and the scores were then put into the software (HSPiP), and the HSP values of PBDB-T could be calculated. The HSP values of PBDB-T were then compared with the HSP values of CB and CN in Hansen space, giving the relative energy distance (RED) value between polymer and solvents, which is a measure of solubility. We believe this method is more accurate in terms of determining solubility than other methods, which only relies on visual inspection of the formation of precipitate during the preparation of the saturated solutions of polymer in solvents of interest.
We understand that there is still a puzzle existing between the lower I0-0/I0-1 ratio of PBDB-T in CN and its slightly lower solubility in the same solvent. Our speculation is the state of aggregation of a polymer in each solvent might not solely depend on the solubility, but also on the polarity of the solvent, or other interactions between polymers and solvents. The relationship between the state of aggregation of polymers and their solubility in a given solvent will be our next research topic.

3. "However, in the PBDB-T:PF5-Y5 binary blend solution, the balance of interactions is different. In CB with added CN, the PBDB-T chains self-aggregate more than in CB." More descriptions should be added to explain the balance of interactions.

We thank the reviewer for pointing this out. The meaning of the balance of interaction is that PBDB-T in solution behaves differently in the presence of PF5-Y5 compared to in absence of PF5-Y5. More specifically, PBDB-T shows less pre-aggregation when it is dissolved in CN, whereas in the PBDB-T:PF5-Y5 binary blend solution with CN as the additive, PBDB-T's self-aggregation is somehow promoted. Based on our observations, we believe that in the PBDB-T:PF5-Y5 binary blend, the role of CN is to mediate the possibly strong interactions between PBDB-T and PF5-Y5 (which leads to the intimate donor:acceptor mixing in the blend film without CN), by making the self-aggregation of PBDB-T more favorable than its interaction with PF5-Y5.
Based on the discussion above, the following contents have been added to the revised manuscript on **page 14**:
"However, in the PBDB-T:PF5-Y5 binary blend solution, the aggregation behavior of PBDB-T is different. More specifically, PBDB-T shows less pre-aggregation when it is dissolved in CN, whereas in the PBDB-T:PF5-Y5 binary blend solution with CN as the additive, PBDB-T's self-aggregation is somehow promoted. … . In another word, the presence of CN shifts the balance of polymer interactions in the binary blend solution, from a possibly strong PBDB-T/PF5-Y5 interaction to a more favorable PBDB-T self-aggregation. As a result, …"

Reviewer: 2

Recommendation: This paper is publishable subject to minor revisions noted. Further review is not needed.

Comments:
The manuscript by Liu et.al investigated the role of the comment additive CN in determining the photovoltaic performance of the all-polymer organic solar cells. The in-situ PL and absorption spectroscopy were used in this work and the results indicated that CN promoted self-aggregation of the donor PBDB-T during the drying process of the blend film. It resulted in improved hole mobility and lower non-radiative recombination voltage loss and higher photovoltaic performance of the PBDB-T:PF5-Y5 organic solar cells. This manuscript deepened our understanding of the role of the solvent additive CN in drying dynamics for all-polymer solar cells. Therefore, I recommend the publication of this manuscript. A few specific suggestions are provided as follows for improving the manuscript.

We thank the reviewer for the positive evaluation of our work.

1. The volume or weight content of the CN in CB is suggested to be indicated in the manuscript.

In this study, we use volume percent to describe the volume content of the CN in solution. The case that was discussed the most in the manuscript is all-polymer solution with 2 v/v% of CN. The preparation of such a solution is described as follows: PBDB-T and PF5-Y5 were mixed with a weight ratio of 1:0.75, the polymer blend was dissolved in CB with a total concentration of 17 mg/mL, followed by adding 2 volume percent of CN into the polymer solution (for example, 20 ul of CN was added into 1mL of polymer solution). We emphases the amount of CN in our polymer solutions is defined by the volume percent on **page 5**, **line 22** in the revised manuscript, and add more details about the preparation of polymer solutions in the revised supporting information (**page S3**)

2. The molecular weight of polymers can greatly affect the device performance of all-polymer solar cells. The molecular weight and PDI value are suggested to be provided in this manuscript.

We thank the reviewer for the suggestion. The molecular weight for PBDB-T in this study is around 45 kDa, with a PDI of 2.0, the molecular weight for PF5-Y5 is around 25 kDa, with a PDI of 2.0. The molecular weight and PDI value have been added in the *Material* section in the revised supporting information (**page S3**).

3. In page 18, the authors draw a conclusion: "the device with 2% CN achieves a ca. 15 mV lower non-radiative energy loss than the reference device, indicating that the crystalized PBDB-T segments might also help to block the nonradiative decay channels of excitons, thus promoting

> more efficient charge separation at the donor/acceptor interface, resulting in a slightly higher VOC in the device with CN." More analysis is needed on the relationship between "block the nonradiative decay channels of excitons" and the "more efficient charge separation at the donor/acceptor interface".

We are sorry for the misunderstanding here. The motivation for conducting the voltage loss calculation is to try to understand the reason behind the increased Voc of the device with CN. Results show that the energy loss through non-radiative recombination was reduced in the device with CN, which leads to our assumption that the crystalized PBDB-T segments might also help to block the nonradiative decay channels. We understand that the reduced recombination at the donor:acceptor interface does not necessarily lead to efficient charge separation, and the relationship between them is not the topic we would like to cover in this work. To minimize the misunderstanding, the content "…, thus promoting more efficient charge separation at the donor/acceptor interface, …" has been removed in the revised manuscript.

Additional Questions:
Urgency: High

Significance: High

Novelty: High

Scholarly Presentation: High

Is the paper likely to interest a substantial number of physical chemists, not just specialists working in the authors' area of research?: Yes
